# Mental health stigma and discrimination in Ethiopia: evidence synthesis to inform stigma reduction interventions

**DOI:** 10.1186/s13033-022-00540-z

**Published:** 2022-06-23

**Authors:** Eshetu Girma, Bezawit Ketema, Tesfahun Mulatu, Brandon A. Kohrt, Syed Shabab Wahid, Eva Heim, Petra C. Gronholm, Charlotte Hanlon, Graham Thornicroft

**Affiliations:** 1 School of Public Health, Addis Ababa University, Addis Ababa, Ethiopia; 2 Department of Psychiatry and Behavioral Sciences, The George Washington University, Washington, DC USA; 3 Department of International Health, Georgetown University, DC Washington, USA; 4 Department of Psychology, University of Zurich, Zurich, Switzerland; 5 Centre for Global Mental Health, Health Service and Population Research Department, Institute of Psychiatry, Psychology and Neuroscience, King’s College London, London, UK; 6 Centre for Implementation Science, Health Service and Population Research Department, Institute of Psychiatry, Psychology and Neuroscience, King’s College London, London, UK; 7 Department of Psychiatry, School of Medicine, WHO Collaborating Centre for Mental Health Research and Capacity Building, Addis Ababa University, Addis Ababa, Ethiopia

**Keywords:** Stigma, Mental health, Discrimination, Ethiopia

## Abstract

**Background:**

People with mental illnesses are at an increased risk of experiencing human rights violations, stigma and discrimination. Even though mental health stigma and discrimination are universal, there appears to be a higher burden in low- and middle-income countries. Anti-stigma interventions need to be grounded in local evidence. The aim of this paper was to synthesize evidence on mental health stigma and discrimination in Ethiopia to inform the development of anti-stigma interventions.

**Methods:**

This evidence synthesis was conducted as a part of formative work for the International Study of Discrimination and Stigma Outcomes (INDIGO) Partnership research program. Electronic searches were conducted using PubMed for scientific articles, and Google Search and Google Scholar were used for grey literature. Records fulfilling eligibility criteria were selected for the evidence synthesis. The findings were synthesized using a framework designed to capture features of mental health stigma to inform cultural adaptation of anti-stigma interventions.

**Results:**

A total of 37 records (2 grey literature and 35 scientific articles) were included in the evidence synthesis. Some of these records were described more than once depending on themes of the synthesis. The records were synthesized under the themes of explanatory models of stigma (3 records on labels and 4 records on symptoms and causes), perceived and experienced forms of stigma (7 records on public stigma, 6 records on structural stigma, 2 records on courtesy stigma and 4 records on self-stigma), impact of stigma on help-seeking (6 records) and interventions to reduce stigma (12 records). Only two intervention studies assessed stigma reduction— one study showed reduced discrimination due to improved access to effective mental health care, whereas the other study did not find evidence on reduction of discrimination following a community-based rehabilitation intervention in combination with facility-based care.

**Conclusion:**

There is widespread stigma and discrimination in Ethiopia which has contributed to under-utilization of available mental health services in the country. This should be addressed with contextually designed and effective stigma reduction interventions that engage stakeholders (service users, service providers, community representatives and service developers and policy makers) so that the United Nations universal health coverage goal for mental health can be achieved in Ethiopia.

**Supplementary Information:**

The online version contains supplementary material available at 10.1186/s13033-022-00540-z.

## Introduction

According to the Global Burden of Diseases, Injuries, and Risk Factors Study 2017, the percentage of global age-standardized Years Lived with Disability (YLD) attributed to mental disorders was 14%, which has had comparably similar levels for nearly three consecutive decades. The study indicated that globally, more than one billion people live with mental disorders and/or substance use disorders. Of these nearly 163 million, 19 million and 175 million people live with major depressive disorder, schizophrenia and substance use disorder, respectively, with depressive disorder being one of the top three leading causes of YLDs in the world [1].

The World Health Organization (WHO) special initiative for mental health highlighted that nearly four out of five people with mental illness (including substance use and neurological disorders) do not receive good quality and affordable mental health care globally [2]. People with mental illnesses are prone to suffer violations of human rights and to experience different forms of stigma and discrimination [2–4]. Even though the problems of stigma and discrimination are known to be universal, their magnitude and severity vary across countries. Thus, low- and middle-income countries (LMICs) have a higher burden compared with high-income countries [5].

Stigma can be conceptualized as experiential (perceived stigma, endorsed stigma, anticipated stigma, received stigma and enacted stigma) or action oriented (public stigma, structural stigma, courtesy stigma, provider-based stigma, and self-stigma). The experiential stigma categories describe how stigma occurs, whereas action oriented stigma forms indicate who/what gives or receives the stigma [6, 7]. Stigma and discrimination against people with mental illnesses has been described as bringing more challenges to affected people than their primary problem of mental illness itself [5].

In Ethiopia the prevalence of schizophrenia, bipolar disorder and major depression is reported as 0.5%, 0.63% and 6.8%, respectively [8, 9]. In a follow up study in a rural part of the country it was highlighted that people with severe mental disorders had a substantially increased risk of death, 13.2% died over a 10 year period, which was almost twice as high as the risk among the general population and amounted to 28 years of life lost per person [10]. Mortality was mostly due to physical health conditions, suggesting that reduced access to healthcare could have contributed, and was shown to be reduced when people had access to mental health care [10, 11]. The health metrics estimate for Ethiopia in 2017 showed that the YLDs for depression, anxiety, bipolar disorder and schizophrenia contributed an estimated 5.32%, 3.60%, 1.23%, and 0.84% of total YLDs, respectively [9].

In low-income countries, like Ethiopia, mental disorders are not considered as life threatening, and have not been given due attention by policy makers and service providers [12]. To address this gap, Ethiopia has implemented scale up of mental health care through integration into primary health care and general medical service since 2012 [13] and planned to have these service in all districts by the end of 2020 [14].

Intervention approaches commonly used to reduce stigma against people with mental disorders are education, contact and protest [15]. The approach of education focuses on enhancing knowledge and awareness, providing factual information in order to reverse myths, wrongly held beliefs and negative attitudes. The approach of contact (also called social or inter-personal contact) can be direct or indirect and aims to enhance interaction and connection between people through sharing of lived experience, recovery stories and challenges so that the audience can overcome fear, build self-esteem and develop empathy. The approach of protest consists of formal objection to stigmatizing beliefs and discriminating behaviors against people with mental illness through advocacy and emphasizing issues of civil rights [7, 16]. Of these approaches, the strategies known to be most effective are education and contact [7, 17–19].

It is very important to consider the variants of stigma and specific groups affected in order to design effective interventions targeting reduction of stigma and discrimination. This could be achieved by understanding the context of the community where the problem has occurred [7, 20, 21]. However, there is a lack of up-to-date reviews that synthesize evidence on mental health stigma and discrimination at the country level in Ethiopia.

The International Study of Discrimination and Stigma Outcomes (INDIGO) is a collaboration of research colleagues in over 40 countries worldwide committed to developing knowledge about mental-illness-related stigma and discrimination, both in terms of their origins and their eradication [22]. INDIGO is coordinated by the Centre for Global Mental Health, Institute of Psychiatry, Psychology and Neuroscience at King’s College London. Since its inception in 2006, the Indigo Network has produced substantial evidence on the impact of stigma and discrimination [3, 23–28] and has now moved its focus towards identifying methods to reduce stigma and discrimination [5, 19, 29]. A detailed description of the program is available on the INDIGO website (http://www.indigo-group.org).

This evidence synthesis was conducted as a part of the UK Medical Research Council-funded INDIGO Partnership research program which aims to develop and test new methods to reduce mental-health related stigma in China, Ethiopia, India, Nepal and Tunisia. The program is aimed at establishing a research collaboration that enables the development of effective, contextually adapted stigma and discrimination reduction interventions, and at carrying out activities to strengthen the scientific understanding of mechanisms of action of stigma processes against people with mental illness. As part of the formative work in the program, this evidence synthesis was conducted to describe mental health stigma and discrimination in Ethiopia.

## Methods

### The country context

Ethiopia is a land-locked country located at the horn of Africa. It is the second most populous country in Africa and has a multilingual and multi-ethnic society. The total estimated population was 108,113,150 in 2020 with a growth rate of 2.56% per annum [30]. The main religious groups are Ethiopian Orthodox Christian 43.5%, Muslim 33.9%, Protestant 18.5%, traditional 2.7%, Catholic 0.7% and other religions 0.6% [31].

The health care system in Ethiopia consists of a mixture of public, private and non-governmental health care sectors [14]. There is a three-tiered health care delivery system containing primary health care units (health post, health center and primary hospital), general hospitals, and specialized hospitals [14]. One of the priority areas of the national health policy stated that appropriate support should be given to the curative and rehabilitative components of health, including mental health [32]. The country has also implemented a national mental health strategy 2012/13–2015/16 [33], launched new national mental health strategy 2021–2025 [34], and adopted the WHO Mental Health Gap Action Programme (mhGAP) [35].

### Design and approach

This evidence synthesis was developed based on the cultural adaptation framework of the INDIGO Partnership program to generate the evidence needed to develop culturally and contextually appropriate anti-stigma interventions. We adopted the framework to extract and synthesize records on explanatory models of stigma, perceived and actual stigma levels, the impacts of stigma on help-seeking, and interventions to reduce stigma and discrimination against people with mental illness.

### Study selection

Records in English language published from 2000 onwards about mental health stigma and discrimination in Ethiopia were eligible for this evidence synthesis. In this study, mental illness was operationalized to describe mental health disorders—psychosis/schizophrenia, bipolar disorder anxiety, depression and substance use disorders in particular. The records that contained content related to the domains of the cultural adaptation framework were included. The domains were: explanatory models of stigma (labels for mental illnesses, symptoms and causes of mental illnesses), forms of perceived and experienced stigma, impact of stigma on help-seeking, and interventions to reduce stigma. Stigma was grouped as public, structural, courtesy, and self-stigma based on the situation where prejudice, stereotypes, discrimination or other stigmatizing characteristics occurred. The grouping was based on the definition provided in other studies of stigma [6, 7, 36]. Accordingly, public stigma is the stigma at general population level in relation to mental health. Courtesy stigma is the stigma experienced by the family/relatives/friends/others that have a relation/proximity to people with mental illness. Structural stigma is the stigma at the level of institutions/organizations/health facilities/social systems due to their policies, laws or regulations regarding mental health. Perceived stigma is the beliefs and expectations that an individual has about the community’s or society’s’ stigmatizing attitudes toward people with mental illness. Experienced stigma refers to their actual encounter with stigmatizing attitudes and discriminating behaviour from the community or society. Self-stigma is internalized stigma when stigmatized individuals accept/approve/apply the mentioned stigma characteristics in their life.

### Search strategy

The literature search was conducted through a range of channels to make sure relevant scientific articles as well as grey literature were not missed. The databases used for the search of grey literature (governmental and non-governmental reports, policy documents and issue papers) were Google search and Google scholar. Whereas, for peer reviewed scientific publications the PubMed database was used. The search terms of this evidence synthesis were not necessarily derived from the conceptual framework, rather we used broader approach to capture any literature related to mental health stigma and discrimination. Thus, the relevant concepts for the search were mental health and psychosocial context, stigma and discrimination, mental health system and interventions. For each concept the search terms were exhaustively determined. Each concept was combined with the search terms with “AND”, whereas the terms in a concept were combined with “OR” in the search engine (Additional file 1: Appendix S1).

### Record synthesis

Each record generated from the databases satisfying the eligibility criteria mentioned above was included. Due to the heterogeneity of identified studies, a narrative synthesis was conducted, structured in accordance with the domains of the conceptual framework.

## Results

**Fig.1:**
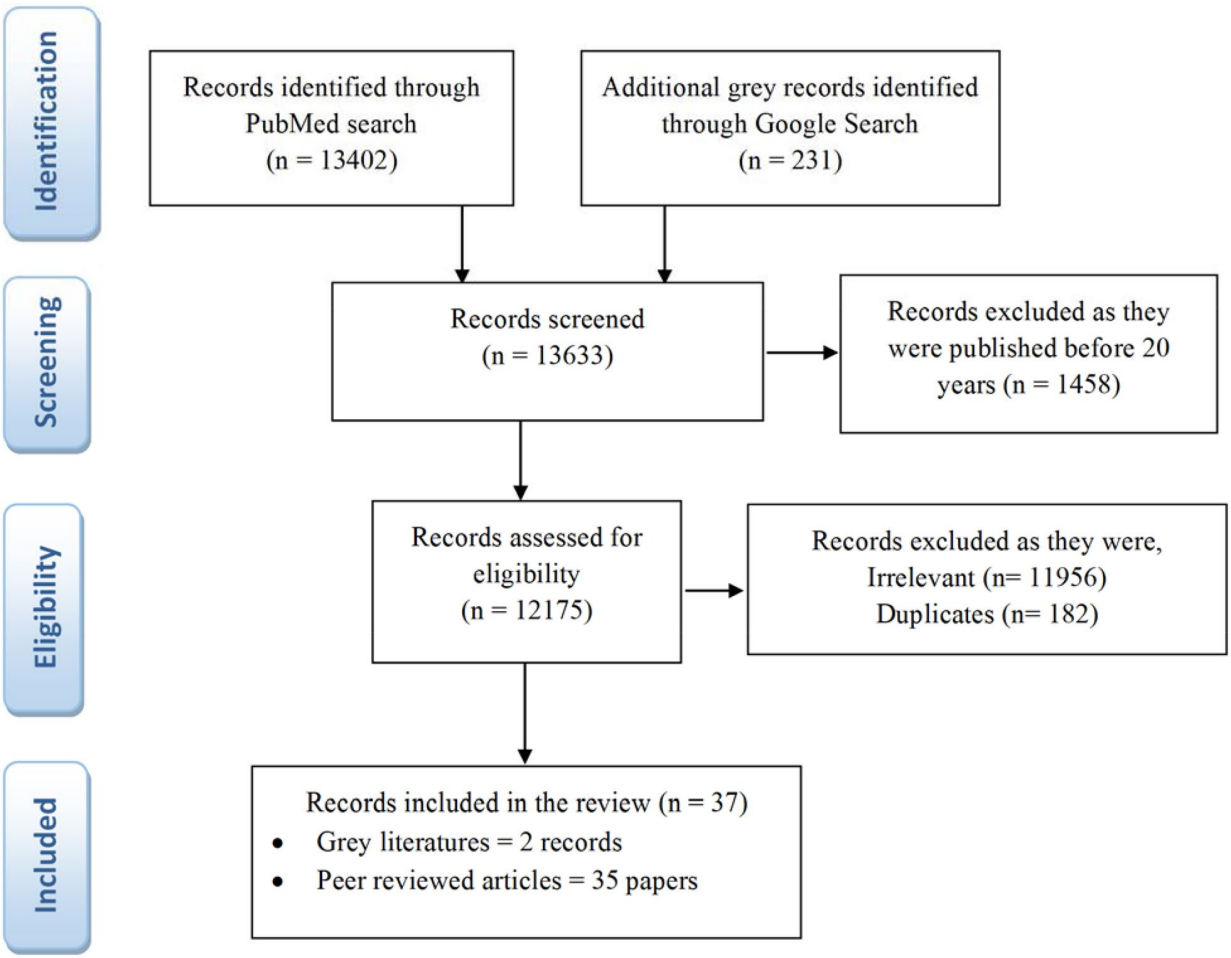
The PRISMA flow diagram

The initial searches from the electronic databases revealed 13,633 records. Records published before 2000 (1458 records) were excluded. Following the screening, 12,175 records were assessed for eligibility and 12,136 records were excluded (duplicates and irrelevant). The remaining 37 records (2 from grey literature and 35 scientific articles) were synthesized (Fig. 1).

Some of the included records were synthesized more than once for different themes. Thus, 7 addressed explanatory models of stigma (3 records on labels and 4 records on symptoms and causes) and 19 described perceived and experienced forms of stigma (7 records on public stigma, 6 records on structural stigma, 2 records on courtesy stigma and 4 records on self-stigma). The synthesis also included 6 records on the impacts of stigma on help-seeking, and 12 records on interventions to reduce stigma (Table 1).

**Table 1:** Synthesized articles

Author, Year and Journal	Literature type	Sample size and study participants	Study area	Measurement tool/scale	Extracted themes from the literature	Main finding of the study
Forthal S. et al. (2019). (BMC Psychiatry)	Quantitative cross-sectional study	300 participants with severe mental illness	Community based study in Sodo district, rural South-central Ethiopia	Semi-structured operational CRITeria for research (OPCRIT) interview, discrimination and stigma scale (DISC12), brief psychiatric rating scale—expanded version (BPRS-E), The 36-item World Health Organization disability assessment schedule (WHODAS-2.0) questionnaire, alcohol use disorders identification test (AUDIT) self-reported version, Oslo 3-item social support scale (OSSS-3)	Service users’ perspectives on public stigma against people with mental illness	High level of experienced discrimination and significant difference was found between urban and rural people with schizophrenia
Tesfaw G. et al. (2020). (International journal of mental health systems)	Quantitative cross-sectional study	423 people with schizophrenia	Institution (Amanuel mental specialized hospital) based study at Addis Ababa, Urban central Ethiopia	OSSS-3, positive and negative syndrome scale (PANSS) and perceived devaluation and discrimination (PDD) Scale	Service users’ perspectives on public stigma against people with mental illness	High perceived stigma among people with schizophrenia
Girma E. et al. (2013). (PLoS One)	Quantitative cross-sectional study	845 participants from the community	Community based study at gilgel gibe field research center, rural Southwest Ethiopia	Community attitudes towards the mentally Ill (CAMI) scale,	Public perspectives on public stigma against people with mental illness	Lower stigma was related with better education and perceived explanatory causes (supernatural/psychosocial/biological) of mental illness
Girma E. et al. (2013). (International journal of mental health systems)	Quantitative cross-sectional study	422 people with severe mental illness	Institution (Jimma university specialized hospital) based study at Jimma town, Urban Southwest Ethiopia	Internalized Stigma of Mental Illness (ISMI) Scale	Service users’ perspectives on self-stigma and interventions to reduce stigma against people with mental illness	There was high feeling of inferiority but less alignment with common stereotypes about people with mental illness
Hadera E. et al. (2019). (Psychiatry Journal)	Quantitative cross-sectional study	384 outpatient service users	Institution (Jimma university specialized hospital) based study at Jimma town, Urban Southwest Ethiopia) based study	Perceived devaluation and discrimination (PDD) scale	Service users’ perspectives on public stigma against people with mental illness	Perceived prevailing stigma among outpatient mental health care service users
Reta Y. et al. (2016). (PLoS One)	Quantitative cross-sectional study	820 participants from the community	Community based study at Jimma town, Urban Southwest Ethiopia	Community attitudes toward the mentally Ill (CAMI) scale	Public perspectives on public stigma against people with mental illness	Prevailing negative attitude against people with mental illness
Hailemariam M. et al. (2017). (Int J Equity Health)	Qualitative study	50 In-depth interviews with service users, care givers and service providers in the facility and 2 FGDs (10 per group) with community service providers	Community based study in Sodo district, rural South-central Ethiopia	Interview guide was developed for countries implementing PRIME	Service users’, caregivers’ and service providers’ perspectives on public stigma against people with mental illness	Financial problems and in accessibility of mental health facility and the medication were the main barriers for the engagement to care’
Abayneh Lempp. et al. (2017). (BMC Psychiatry)	Qualitative study	39 interviews with service users, care givers, managers and policy makers/planners/service developers at national and regional level	Community based study at Butajira (rural central part of Ethiopia) and with national representatives	Interview guide was developed for Emerging mental health systems in low- and middle-income countries (Emerald) cross-country study	Service users’, caregivers’ and service providers’ perspectives on public stigma, structural stigma and intervention on stigma	Despite the increasing access of mental health care, there were barriers in relation to stigma and awareness
Hanlon C. et al. (2017). (International journal of mental health systems)	Qualitative study	17 in-depth interviews with national and regional policy makers, service developers and district level health office managers	Community based study in Sodo district, rural South-central Ethiopia	Interview guide was derived from the adapted health system governance (HSG) evaluation framework	Policy makers’ and service developers’ perspectives on structural stigma	There are improved government support and commitment to scale-up mental health care. however, the leadership and coordination among different administrative levels needs to be strengthened in addition to addressing awareness problems
Hailemariam M. et al. (2019). (International Journal of Mental Health Systems)	Quantitative cross-sectional study	369 participants with probable cases of severe mental illness	Community based study in Sodo district, rural South-central Ethiopia	Barriers to access to care evaluation (BACE-3), short explanatory model interview (SEMI), WHO 12 item Disability Assessment Schedule (WHODAS 2.0), Oslo-3 scale, alcohol use disorders identification test (AUDIT), discrimination and stigma scale (DISC-12), Brief physical impairment rating checklist (BPIRC), Washington Group’s disability measure and Work, Family and Well-being (WFW) scales	Service users’ perspectives on structural stigma, interventions to reduce stigma and impact of stigma on help-seeking of people with severe mental illness	Integrating mental health care into primary care highly improves the level of equitable contact coverage
Abayneh S. et al. (2020). (International journal of mental health systems)	Qualitative study, theory of change (ToC) development	31 participants (4 psychiatrists, 3 researchers, 24 stakeholders involved in service planning)	Community and institution-based study at sodo district (rural south-central Ethiopia) and Addis Ababa (urban central Ethiopia) respectively	ToC Maps, workshop and meeting minutes	Service providers’ and service developers’ and administers’ perspectives on structural stigma and interventions to reduce stigma	In order to mobilize and empower service users and caregivers for mental health system improvement, the service user and caregiver program level was the main intervention component identified in the ToC
Girma E. et al. (2014). (Journal of multidisciplinary healthcare)	Quantitative cross-sectional study	422 participants who were caregivers	Institution (Jimma University Specialized hospital) based study at Jimma town, Urban Southwest Ethiopia) based study	Questionnaire adopted from WHO family interview schedule stigma items and other literatures	Care givers’ perspectives on self-stigma due to family members’ mental illness	Even though the care givers’ self-stigma was found to be low, it might have an impact on help seeking behavior of people mental illness
Girma E. et al. (2014). (BMC international health and human rights)	Quantitative cross-sectional study	845 participants from the community	Community based study at gilgel gibe field research center, rural Southwest Ethiopia	Questionnaire adopted from devaluation of consumer families scale and other literatures	Care givers’ perspectives on public stigma due to family members’ mental illness	There was moderate level of perceived public stigma against family members of PWMI and it was negatively correlated with perceived symptoms and explanatory causes (supernatural/psychosocial/biological) of mental illness
Assefa D. et al. (2012). (BMC Psychiatry)	Quantitative cross-sectional study	212 individuals with schizophrenia	Institution (Amanuel Mental Specialized Hospital) based study at Addis Ababa, Urban Central Ethiopia	Internalized stigma of mental illness (ISMI) Scale and diagnostic and statistical manual of mental disorders, fourth edition (DSM-IV)	Service users’ perspectives towards self-stigma among people with schizophrenia	Internalized stigma was indicated to be the prevailing problem among people with schizophrenia as nearly half of them had the problem
Bifftu B. B. et al. (2014). (BMC Psychiatry)	Quantitative cross-sectional study	411 individuals with schizophrenia	Institution (Amanuel mental specialized hospital) based study at Addis Ababa, Urban Central Ethiopia	Internalized stigma of mental illness (ISMI) scale	Service users’ perspectives towards self-stigma among people with schizophrenia	Low stigma resistance was reported by nearly half of the participants with schizophrenia
Zewdu S. et al. (2019). (Substance abuse treatment, prevention, and policy)	Quantitative cross-sectional study	1500 adults from the community	Community based study in Sodo district, rural South-central Ethiopia	Alcohol use disorder identification tool (AUDIT), patient health questionnaire (PHQ-9) for depression, WHO composite international diagnostic interview (CIDI), WHO disability assessment schedule (WHODAS) version 2.0, with 12 items, list of threatening experiences (LTE) questionnaire, 3-item Oslo social support (OSS) questionnaire and internalized stigma of mental illness inventory (ISMI)	Service users’ perspectives towards self-stigma among people with alcohol use disorder	The twelve-month prevalence of alcohol use disorder was 13.9% and most of whom didn’t seek medical help and had high internalized stigma
Asher L. et al. (2017). (Globalization and Health)	Qualitative study (RISE study)	35 participants (5 in-depth interviews and 5 focus group discussions) composed of people with schizophrenia, their caregivers, community leaders and primary and community service providers	Community based study in Sodo district, rural South-central Ethiopia	Used topic guide as part of rehabilitation intervention for people with schizophrenia in Ethiopia (RISE) project	Care givers’ perspectives on the impact of stigma on help-seeking behavior of people with mental illness	Physical restraint of people with schizophrenia was common practice which could restrict them from attending the needed medical care
Fekadu A. et al. (2019). (BMC Psychiatry)	Quantitative cross-sectional study	300 participants with psychosis	Community based study in Butajira, rural central part of Ethiopia	The Butajira treatment gap questionnaire (TGQ), brief psychiatric rating scale- expanded version (BPRS- E), WHO disability assessment schedule (WHODAS 2.0), Oslo 3 social support scale (OSS), Operational Criteria for Research (OPCRIT)	Service users’ perspectives towards the impact of stigma on help-seeking behavior of people with mental illness	Nearly six out of ten participants had current access gap to biomedical care, which could affect their work function and might experience discrimination
Souraya S. et al. (2018). (Globalization and Health)	Qualitative study	18 in depth interviews (people with schizophrenia, caregivers, service providers and officials) and 2 FGDs with community- based rehabilitation workers	Community based study in Sodo district, rural South-central Ethiopia	Used topic guide as part of rehabilitation intervention for people with schizophrenia in Ethiopia (RISE) project	Service users’ and caregivers’ perspectives towards the impact of stigma on help-seeking behavior of people with mental illness	It is not common practice to involve service users and care givers in decision making towards the treatment options
Girma E. and M. Tesfaye (2011). (BMC Psychiatry)	Quantitative cross-sectional study	384 participants with psychosis	Institution (Jimma University Specialized Hospital) based study at Jimma town, Urban Southwest Ethiopia	Questionnaire was developed based on WHO Encounter Form for Pathways to care and good’s pathway model	Service users’ perspectives towards the impact of stigma on help-seeking behavior of people with mental illness	There was significant delay in treatment seeking behavior among people with psychosis
Tsigebrhan R. et al. (2014). (Schizophrenia Research)	Quantitative comparative cross-sectional study	201 participants with severe mental illness	Community based study in Butajira, rural central part of Ethiopia	Questionnaire was prepared based on the macarthur violence interview, modified version of the historical, clinical and risk management (HCR-20) scale and other literatures	Service users’ perspectives towards the impact of stigma on help-seeking behavior of people with mental illness	There significantly higher level of violence and violence victimization among people with severe mental illness which alarms the need improvement in mental health care access
Tirfessa K. et al. (2020). (Tropical medicine & international health)	Quantitative controlled before-after study	239 participants with severe mental illness and 273 participants as control households	Community based study in Sodo district, rural South-central Ethiopia	Household food insecurity access scale (HFIAS), discrimination and stigma scale‐12 (DISC‐12), WHO Disability Assessment Schedule (WHODAS) 2.0 12‐item version, longitudinal interval follow‐up evaluation‐range of impaired functioning tool (LIFE‐RIFT) and 24‐item brief psychiatric rating scale‐expanded version (BPRS‐E)	Service users’ perspectives towards interventions to reduce stigma against people with mental illness	The improvement in access to mental health care led to improvement in the households’ food security of more than half of the households with people with severe mental illness
Habtamu K. et al. (2018). (Social psychiatry and psychiatric epidemiology)	Quantitative cross-sectional study	324 participants with severe mental illness	Community based study at Butajira, rural central part of Ethiopia	WHO Disability Assessment Schedule (WHODAS-2.0), Butajira functioning scale (BFS), brief psychiatric rating scale (BPRS-E), Composite International Diagnostic Interview (CIDI) substance abuse module, Mini International Neuropsychiatric Interview (MINI) Suicidality Scale, Antipsychotic Side effects Checklist (ASC), Life Chart Schedule (LCS), Internalized Stigma of Mental Illness (ISMI) scale	Service users’ perspectives towards interventions to reduce stigma against people with mental illness	Functional impairment was determined by symptom severity, poverty, medication side effects, and internalized stigma among people with severe mental illness
Asher L. et al. (2015). (PloS One)	Qualitative study	Two consecutive workshops with 8 mental health experts and 12 community leaders respectively, 16 in-depth interviews and 5 FGDs with service users (people with schizophrenia), caregivers and service providers	Intervention development work in sodo district, rural South-central Ethiopia	workshop notes, site visits and in-depth consultation, data extraction and interview guide	Service users’, care givers’ and providers’ perspectives towards interventions to reduce stigma against people with mental illness	People with schizophrenia have challenges in maintaining work function, social and family role and experienced stigma which are perceived to be addressed with culturally appropriate, acceptable and feasible community-based rehabilitation intervention
Asher L. et al. (2018). (BMC Psychiatry)	Follow up mixed method (qualitative and quantitative) pilot study	31 individuals (people with schizophrenia, caregivers, community members and service providers)	Community based pilot study at Sodo district, rural South-central Ethiopia	Used topic guide, data extraction form, and measurement scales; Discrimination and Stigma Scale-12 (DISC-12) subscale 1, Alcohol Use Disorders Identification Test (AUDIT), Patient Health Questionnaire-9 (PHQ-9), Involvement Evaluation Questionnaire (IEQ), WHO Disability Assessment Schedule (WHODAS) 2.0 and Clinical Global Impression (CGI)	Service users’, care givers’ and providers’ perspectives towards interventions to reduce stigma against people with mental illness	Community-based rehabilitation intervention was proven to be acceptable and feasible approach which should be used with the existing facility-based care for people with schizophrenia
Hanlon C. et al. (2019). (Epidemiology and psychiatric sciences)	Quantitative interventional cohort study (PRIME study)	245 participants with severe mental illness	Institution based study at Sodo district, rural South-central Ethiopia	Brief Psychiatric Rating Scale, expanded version (BPRS-E), WHO Disability Assessment Schedule (WHODAS), ‘unfair treatment’ subscale of the Discrimination and Stigma Scale-12 (DISC-12), Alcohol Use Disorder Identification Test (AUDIT), locally validated version of the Patient Health Questionnaire (PHQ-9) and three-item oslo social support scale (OSS-3)	Service users’ perspectives towards discrimination and interventions to reduce stigma against people with mental illness	Nearly less than one third of participants had received the minimum required treatment available at district level. The follow up has revealed that those who received the treatment had significant improvement in severe mental illness symptoms, disability measures, depression symptoms, discrimination, restraining, alcohol use disorder and suicidal attempt
Asher L. et al. (2021). (Lancet preprint)	Quantitative study (cluster-randomized controlled trial)	In intervention and control arm, 79 and 87 participants with schizophrenia respectively	Community based study at Sodo district, rural South-central Ethiopia	WHO Disability Assessment Schedule (WHODAS 2·0), Brief Psychiatric Rating Scale-Expanded (BPRSE) score, Clinical Global Impression (CGI), Butajira Functioning Scale score, Chart Schedule, Adapted Client Service Receipt Inventory (CSRI), Discrimination and Stigma Scale-12 (DISC-12), Involvement Evaluation Questionnaire (IEQ) and Patient Health Questionnaire-9 (PHQ-9)	Service users’ perspectives towards interventions to reduce stigma against people with mental illness	After 12 months of community based rehabilitation intervention, disability was effectively reduced. However, it didn’t found evidence on reduction stigma

### Explanatory models of stigma

#### Mental illness labels

An experience shared by an Ethiopian living abroad and on a temporary visit in the country, stated that she heard the terms “insane” and “crazy” rather than “mentally ill” when national media made announcements for missing people with mental illness [37]. Similarly a clinical psychologist claimed that her clients were repeatedly using the term “I must be really crazy” [38].

In a qualitative study among mental health providers, most respondents perceived mental health as a basic component of a person’s wellbeing. Some of the participants reported mental health as inter-connectedness of psychological, physical and emotional wellbeing. Others described mental health as being free from mental illness or ability to perform daily functions, and a similar number of respondents described it in relation to behaving in an acceptable way in a society or workplace [39].

#### Perceived causes and symptoms of mental illnesses

In a focus group discussion among religious leaders, health workers and community participants in the northern part of the country, the perceived common causes of mental illness pointed out were; controlled by evil spirit due to violation of God’s rules, attack by devil spirit, grief from loss of a loved one, poverty, too much thinking, and substance use [40]. In the same study, most participants characterized people with mental illness as aggressive and violent physically. They also mentioned the fear of danger imposed by people with mental illness as a cause of stigma. In a study among health workers, community members and traditional healers, participants perceived that the expression of negative emotions could help to recognize depression and anxiety, whereas bizarre or unusual behavior could help to recognize psychosis [41].

A study in a rural part of the country revealed gender-related differences. Community responses to individuals with severe mental illness were reported to vary according to gender and visible symptoms in public spaces. Participants described feelings of sympathy and pity towards women who were visibly ill in public spaces. The reason for being more compassionate was due to considering the impact of the illness on a woman’s domestic role, and the ability to have and care for her family. According to this study, diagnoses of mental illness in women were often kept secret or concealed within the home. Women with mental illnesses were still responsible for their family and household obligations and received less social support from their family. This made women vulnerable to neglect, sexual exploitation, and abuse [42].

According to one study among health workers, community members and traditional healers, the perceived causes of mental illnesses were related to psycho-cultural inappropriateness (culturally unacceptable behaviors, violating societal taboos, unfavorable attitudes, and lack of balance towards socially acceptable values), religious/spiritual factors, social difficulty, behavioral disturbance, cognitive-emotional impairment, disaster and economic deprivation, difficulties in adapting to environmental changes, substance abuse, and physical/medical conditions [41]. In a population-level study of mental distress in a rural part of the country, psychosocial stressors/stressful life events were predominant and strongly related with common mental illnesses [43].

### Perceived and experienced forms of stigma

#### Public stigma

According to a qualitative study which involved 26 mental health practitioners or educators, participants perceived community stigma as soon as individuals with severe mental illness were seen outside their homes, regardless of gender [39]. In a population-based study among people with severe mental illness, 63.3% of 300 participants had experienced discrimination in the previous year, mainly by being avoided or shunned because of mental illness [44]. An institution-based study in an urban setting reported similar findings of perceived stigma (62.6%) among 423 people with schizophrenia [45].

A study that assessed the community’s attitude towards mentally ill in the south-west part of Ethiopia has shown that rural residents had significantly higher scores for perceived stigma as compared to urban residents. It was indicated that there was a negative association between degree of perceived stigma and the level of education. Those with higher scores of perceived supernatural causes or perceived psychosocial and biological causes had lower stigma scores [46]. In another similar study in the general public, there were higher perceived stigma scores towards people with mental illness among those with government or private employment, who were married, or had completed secondary school as compared to housewife, single, or completed primary education respectively [47].

In a facility-based study of south-west Ethiopia, the prevalence of perceived high and low stigma was 51% and 44% respectively among 384 people with mental illness seeking outpatient mental health care. In this study, substance use history, lack of family support and medication side effects were associated with higher perceived stigma [48]. In a qualitative study in a rural region of Ethiopia, the chance of marrying was perceived to be lower for individuals (both genders) with severe mental illness, because of the difficulty in finding a partner and starting a family due to perceived dangerousness and the prevalent stigma of mental illness. Men had a better chance of finding a partner after treatment than women, as impaired functioning due to severe mental illness was perceived to affect the ability to cook, care and clean among women. Women also have a higher risk of being separated or divorced due to a mental illness. If a woman was unable to perform the expected family role, the participants mentioned that she might be sent back to her family. For men, the risk of being abandoned due to mental illness was much lower [49].

#### Structural stigma

There are only few studies assessing structural stigma in Ethiopia. One of these is a qualitative study that included representatives from national and regional planners, service developers and policy makers of Ethiopia. The participants mentioned that there was government commitment and support for integration of mental health services at the primary health care level. Nevertheless, it was described that there were problems with regards to awareness, transparency, stakeholder involvement and coordination in mental health care planning and decision making. In addition, shortage of medication supplies and lack of community mobilization for mental health, and inadequate health management information system indicators needed for monitoring were among the structural barriers for mental health care scale-up in Ethiopia [50]. In a related community-based study among people with severe mental illness, those who were never engaged with mental health care perceived the cost of treatment as one of the barriers, and the fear of being treated differently was also mentioned by some participants [51].

In a qualitative study involving service users, caregivers and service providers, it was mentioned that the longer duration of treatment and inaccessible nature of the facilities providing the care were identified as the main barriers for engagement with mental health care, in addition to financial problems [52]. Additionally, people with schizophrenia and their caregivers had limited capacity to make a decision as the treatment options were limited, not feasible and difficult to access [53].

In a study about barriers and facilitators of service user involvement in mental health system strengthening, participants perceived prevailing negative attitudes towards mental health and towards people with mental illness among service providers, managers and policy developers. Lack of prioritization of mental health in policy development, and a lack of legislation, or culture of working with people with mental illness as partners were additional structural barriers for involvement of service users in policy drafting and planning, service development and quality monitoring [54]. A similar qualitative study on service user involvement in mental health system strengthening found unreliable medication supplies as a source of dissatisfaction among service users and service providers. Additionally, problems with regards to communication between service users and providers and a lack of evidence-based information about the medication options were also mentioned as barriers for involvement [55].

#### Courtesy stigma

Community-based studies in the south-west part of Ethiopia indicated that caregivers of people with mental illness isolated themselves from social life due to fear of public stigma and discrimination [56, 57]. They felt shame or embarrassment about the family member’s illness. They were worried that other people would find out about the illness and preferred to hide and keep it as a secret from other people and when visiting social events with their family member. It was indicated that family stigma was found to be moderately high and that being a rural resident was significantly associated with higher stigma scores. However, having a better explanation of mental illnesses was associated with decreased stigma.

#### Self-stigma

A study in a specialized psychiatric hospital in Addis Ababa indicated internalized stigma as the prevailing problem among people with schizophrenia. Nearly half of 212 participants had moderate to high self-stigma scores [58]. In another study from the same setting, around half of the participants reported low stigma resistance (the ability to remain unaffected by the stigma) [59].

An investigation from southern rural Ethiopia indicated that more than two thirds of people with alcohol use disorder had internalized stigma [60]. In a similar study, higher self-stigma scores were related to being female, having a history of traditional treatment, and a higher perceived supernatural explanation of mental illness [61].

#### Impact of stigma on help-seeking

There can be delayed treatment seeking behavior among people with mental illness. In a study among people with alcohol use disorder in a rural south-central part of Ethiopia, more than 80% of the participants did not receive any medical help for their illness [60]. Another study in south-west Ethiopia has showed that 65.1% of participants came for treatment after significant delays and more than half of them had tried traditional options (religious or herbal) before their arrival to the health facility. Those with symptoms like abdominal pain and headache were more likely to seek care early [62].

According to a qualitative study among mental healthcare providers [39], the majority of participants perceived that people should rely mainly on interventions from religious institutions to address mental health and wellbeing issues. This factor, in addition to inaccessibility of mental healthcare, is related to the high stigma around seeking modern mental health interventions, as opposed to the normative process of seeking help from the church. In another community-based study on plans to improve mental health care access among people with severe mental illness, some respondents perceived stigma to be one of the barriers to non-engagement, although they did not think it would prevent attendance for most people [51].

The practice of involving people with mental illnesses in decision making on treatment options is not common. A study in a rural part of the country among people with schizophrenia revealed that the main decision makers were the caregivers and health workers. People with mental illnesses were merely consulted with regard to their treatment options, which indicated the prevailing pervasive stigma.

A study in a rural setting of Ethiopia found that people with severe mental illness were more likely to be victims than perpetrators of violence, but that perpetration of violence was more common than in the general population and reflected a lack of access to mental health care [63]. A study in a rural central part of the country among people with psychosis showed that nearly half had lifetime or current gaps in accessing biomedical care, and from those who received care, more than two thirds had minimally inadequate care [64].

#### Interventions to reduce stigma

A research PRogramme for Improving Mental HealthcarE (PRIME), which evaluated the impact of integrated mental health care in primary care in rural Ethiopia highlighted that, in addition to enhancing mental health care access and work functioning of people with severe mental illness, reducing discrimination could be important to reduce household food insecurity [65]. In another report of the same programme, although only less than one third of participants had received the minimum required treatment available at district level, there were significant improvements in clinical and social outcomes of people with severe mental illness. It was revealed that those who received the treatment had significant improvement in severe mental illness symptoms, disability measures, depression symptoms, discrimination, restraint, alcohol use disorder and suicidal attempt [66].

Studies conducted to develop and evaluate the community-based rehabilitation intervention (CBR) for people with schizophrenia in Ethiopia (RISE) have indicated that the approach could be cost-effective and acceptable, if it could be used with the available care in the facility and considering the setting of the community. This intervention approach is composed of home visits by trained (about the intervention) representatives from the community, community mobilization, and family support groups [67, 68]. As per findings of 12-month cluster randomized controlled trial from RISE, disability was effectively reduced following a combination of CBR and task-shared facility-based care for people with schizophrenia. However, there was no evidence that CBR had an impact on discrimination as measured by Discrimination and Stigma Scale-12 (DISC-12) [69].

In research to inform the development of care for people with severe mental illness, stakeholders mentioned that the interventions should focus on re-establishing the living conditions of people with severe mental illness. They described that it could be challenging and might need a longer duration to achieve daily necessities, as poverty is the underlying condition for them. The innovative care for chronic conditions framework was adapted for the rural setting considering the importance of tackling stigma in the community, and using the role and resources of the families, community workers, traditional and religious leaders in addition to biomedical care [70].

It was indicated that awareness-raising component was included as a strategy for reduction of stigma and discrimination at the community level in rural Ethiopia [71]. Thus, using the community health workers especially health extension workers in rural Ethiopia could be important to enhance the community awareness due to their proximity to the community. Engaging the service users and caregivers in the awareness raising training could be effective in reducing public stigma [55].

An assessment of health workers’ beliefs and attitudes in rural Ethiopia showed that after awareness enhancing training was provided to health workers on mental and developmental disorders, the level of stigmatizing attitudes decreased as indicated by lower scores on the social distance scale [72]. In one study it was suggested that empowerment with psychosocial interventions and addressing drug side effects could be helpful in reducing self-stigma among people with mental illnesses [61]. Another qualitative study in a rural setting has suggested that in addition to enhancing the access to mental health care, community outreach and mental health awareness raising programs could be an approach to reduce stigma and improve social outcomes of people with severe mental illness [49]. Similarly, interventions targeting the prevention of internalized stigma could reduce the functional impairment of people with severe mental illness [73].

## Discussion

In our evidence synthesis we found that, in Ethiopia, people with mental illnesses were exposed to prevailing public stigma [39, 44–48, 52, 54], structural stigma [50, 51, 54, 55, 74], courtesy stigma [56, 57] and self-stigma [58–61]. The fear of danger from aggressive and violent behavior was claimed as a reason for the stigma [40], which was also a reason mentioned in Asia [75]. Even though it is common to show sympathy for women with mental illnesses, it could be very challenging for them to attain their normal life [42], especially in finding a partner and establishing a family [49]. Additionally, there was low stigma resistance [59] and delayed treatment seeking [62] for mental illnesses, as it was common to believe in divine and traditional healing systems [39–41].

We did not find any intervention studies with the main aim of reducing stigma and discrimination in our setting. However, there were two studies, the RISE [69] and PRIME [66] studies, which had secondary outcomes of stigma reduction. The RISE study did not find evidence of reduction of discrimination by community-based rehabilitation. However, the PRIME results showed reduced discrimination, that is, increased access to effective mental health care. Thus, the following suggestions were also provided based on the findings of other studies with different primary objectives. In order to reduce stigma, economic empowerment [49] was suggested, as poverty was claimed to be a cause of stigma in Africa [21]. There were also initiatives underway to use social contact interventions [55]. However, awareness raising [46, 49, 61] was the main intervention recommended. This might be the appropriate approach, as evidence [7, 17] supports education as an effective approach to address mental illness stigma. Although outcomes were not available at the time of this review, stigma reduction among primary care workers through social contact with service has been piloted in Ethiopia, based on the REducing Stigma among HealthcAre providErs (RESHAPE) intervention developed in Nepal. The intervention components of RESHAPE were service user recovery stories and social contact; aspirational figures; myth busting; stigma didactics; and collaboration [76, 77]. As was stated in the vision of the WHO mental health strategy [4], people with mental illness should attain a full range of health and social services free from stigma and discrimination. However, achieving this might be challenging in Ethiopia, as mental illness was not accepted as culturally appropriate [21] and the terms used to describe the problem [37, 38] were stigmatizing. Besides, evidence [78] has indicated that all countries could fail to achieve the universal health coverage (UHC) goal for mental illnesses, if they were to continue with systems having stigmatizing barriers in relation to mental health services budgets.

The WHO has emphasized that mental health must be an integral part of UHC with adequate and quality services [2]. However, inaccessible mental health care services especially for people who are living in rural areas of the country is still a challenge [63, 79]. Another health system factor which contributes to prevailing stigma in the country is related to mental health workforce and awareness problems. Ethiopia has five times less mental health workforce staff than the global average, which places the country even below the average of low-income countries [80–82] and WHO recommendations of access to mental health care [4, 79]. In addition, there are gaps in involving service users in mental health care—they have limited opportunities to make a decision on treatment options [53].

Stigma is pervasive and affects communities, healthcare providers, planners and policy-makers. There is low mental health awareness among service users [83], service providers [84, 85] and policy makers [50], accompanied by under-investment in mental health care [86] which might further contribute to the prevailing structural stigma. Even though there are opportunities [86], it could be difficult for Ethiopia to achieve UHC goal for mental illnesses, as it is required to increase service coverage by 50% for severe mental health conditions [2]. It should be noted that, in addition to monitoring the implementation of the plans, if the capacity building activities [87] and context specific strategies [86] could be applied effectively, including reduction of structural stigma, there is still hope of achieving the UHC goal for mental health disorders.

There is limited availability of evidence-based mental health care in Ethiopia, resulting in a high treatment gap [64]. From an international review [88], stigma reduction interventions were known to improve the lives of people with mental illnesses as indicated by a multilevel model containing the framework of interventions to reduce excess mortality. Addressing mental health stigma could also be helpful to achieve UHC for mental illness as per the evidence from a global review [78]. Therefore, evidence generated from this synthesis could be used as an up-to-date summary of the available evidence regarding Ethiopia’s mental health stigma context. However, we acknowledge that this evidence synthesis should be supported by nationally representative primary data.

## Conclusion

This study showed the prevailing public, structural, courtesy forms of stigma and self-stigma in different parts of Ethiopia, which may adversely affect quality of life and help-seeking behaviour by people with mental illness. Achieving the UHC goal by 2030 may not be realistic in the country without integrating mental health stigma-reduction approaches into efforts to scale up mental health care. However, it can be possible to achieve the goal if context specific strategies are developed, implementation of the plans are monitored, the available opportunities used effectively, and if capacity building activities are enhanced.

Moreover, there should be engagement of mental health service stakeholders (service users, caregivers, religious institutions, community leaders, local social services, primary care providers, specialists and policy makers) in the process of designing or adapting stigma interventions, so that these could be contextually realistic, effective and sustainable.

## Supplementary Information

**Additional file 1: Appendix S1** The concepts and terms used for electronic database search.

## Data Availability

The datasets used and/or analyzed during the current study are available from the corresponding author on reasonable request.
