# Supplementary material for: Mental health stigma and discrimination in Ethiopia: evidence synthesis to inform stigma reduction interventions
Source: Int J Ment Health Syst. 2022 Jun 23;16:30. doi: 10.1186/s13033-022-00540-z (PMC9219139; doi:10.1186/s13033-022-00540-z)
Supplement: Supplementary file 1 — Additional file 1: Appendix S1 The concepts and terms used for electronic database search. [file 13033_2022_540_MOESM1_ESM.docx]

Appendix S1 The concepts and terms used for electronic database search

| Search concepts | Search terms |
| --- | --- |
| Mental Health and Psychosocial Context | mental health, mental disorders, depression, affective disorders, mood disorders, anxiety, psychosis, schizophrenia, common mental disorders, severe mental disorders, bipolar disorders, substance use disorders |
| Stigma and Discrimination | stigma, discrimination, stereotypes, beliefs, attitudes, prejudice, social perception, social distance |
| Mental Health System | mental health policy, mental health system, mental health services, primary health care, mental health care, specialised care, lay workers, health workers, nurse, health officer, doctor, social worker |
| Interventions | intervention, anti-stigma, social contact, education, training, help seeking, treatment |
| Country | Ethiopia |
